# Supplementary material for: Mutational Spectrum of the ABCA12 Gene and Genotype–Phenotype Correlation in a Cohort of 64 Patients with Autosomal Recessive Congenital Ichthyosis
Source: Genes (Basel). 2023 Mar 15;14(3):717. doi: 10.3390/genes14030717 (PMC10048568; doi:10.3390/genes14030717)
Supplement: Supplementary file 1 [file genes-14-00717-s001.zip › genes-2199576-supplementary.pdf]

Supp. Table S1: classification of the novel mutations in *ABCA12*

Mutation		gnomAD	ClinVar	ACMG classification
c.70-2A>G	p.?	n/a	n/a	pathogenic (PVS1, PM2, PM3, PP3)
c.646_647del	p.(Thr216Profs*19)	n/a	n/a	pathogenic (PVS1, PM2, PM3)
c.1221dup	p.(Ser408Ilefs*9)	n/a	n/a	pathogenic (PVS1, PM2, PM3)
c.1792_1801del	p.(Gln598Glyfs*14)	n/a	n/a	pathogenic (PVS1, PM2, PM3)
c.2194C>T	p.(Gln732*)	n/a	n/a	pathogenic (PVS1, PM2, PM3)
c.2251_2252delinsT	p.(Gly751Serfs*8)	n/a	n/a	pathogenic (PVS1, PM2, PM3)
c.2341T>A	p.(Cys781Ser)	n/a	n/a	likely pathogenic (PM1, PM2, PM3, PP3)
c.2486dup	p.(Arg830Glufs*16)	n/a	n/a	pathogenic (PVS1, PM2, PM3)
c.2509del	p.(Glu837Lysfs*14)	n/a	n/a	likely pathogenic (PVS1, PM2, PM3)
c.2968A>G	p.(Lys990Glu)	n/a	1x uncertain significance	likely pathogenic (PM1, PM2, PM3, PP3)
c.2972_2988del	p.(Thr991Lysfs*31)	n/a	n/a	pathogenic (PVS1, PM2, PM3)
c.3260dup	p.(Leu1088Alafs*4)	n/a	n/a	pathogenic (PVS1, PM2, PM3)
c.3276del	p.(Asp1093Thrfs*8)	n/a	n/a	pathogenic (PVS1, PM2, PM3)
c.3452T>A	p.(Phe1151Tyr)	0x homo, 3x het, MAF 0.001%	n/a	likely pathogenic (PM1, PM2, PM3, PP3)
c.3758T>C	p.(Leu1253Pro)	n/a	n/a	likely pathogenic (PM1, PM2, PM3, PP3)
c.3829+3A>G	p.?	n/a	n/a	likely pathogenic (PM2, PM3, PP3)

c.3977del	p.(Ser1326Ilefs*41)		n/a	pathogenic (PVS1, PM2, PM3)
c.4412A>G	p.(His1471Arg)	0x homo, 2x het, MAF 0.0007%	n/a	likely pathogenic (PM1, PM2, PM3, PP3)
c.4468T>C	p.(Ser1490Pro)	n/a	n/a	likely pathogenic (PM1, PM2, PM3, PP3)
c.4512_4515del	p.(Glu1504Aspfs*39)	n/a	n/a	pathogenic (PVS1, PM2, PM3)
c.4514C>T	p.(Pro1505Leu)	0x homo, 1x het, MAF 0.0004%	n/a	likely pathogenic (PM1, PM2, PM3, PP3)
c.4540C>T	p.(Arg1514Cys)	n/a	n/a	pathogenic (PM1, PM2, PM3, PM5, PP3)
c.4601C>T	p.(Thr1534Met)	0x homo, 3x het, MAF 0.001%	n/a	likely pathogenic (PM1, PM2, PM3, PP3)
c.5046_5050del	p.(Lys1682Asnfs*13)	n/a	n/a	pathogenic (PVS1, PM2, PM3)
c.5787T>G	p.(Tyr1929*)	0x homo, 2x het, MAF 0.0008%	1x pathogenic	pathogenic (PVS1, PM2, PM3, PP5)
c.5878C>T	p.(Arg1960*)	n/a	n/a	pathogenic (PVS1, PM2, PM3)
c.5939+1G>A	p.?	0x homo, 4x het, MAF 0.002%	n/a	pathogenic (PVS1, PM2, PM3, PP3)
c.6356T>G	p.(Val2119Gly)			likely pathogenic (PM1, PM2, PM3, PP3)
c.6393G>T	p.(Pro2131=)	0x homo, 6x het, MAF 0.002%	n/a	pathogenic (PVS1, PM2, PM3, PP3)
c.6722_6723del	p.(Arg2241Ilefs*4)	n/a	n/a	pathogenic (PVS1, PM2, PM3)
c.7006T>G	p.(Cys2336Gly)	n/a	n/a	likely pathogenic (PM1, PM2, PM3, PP3)
c.7222C>T	p.(Pro2408Ser)	0x homo, 1x het, MAF 0.0004%	n/a	likely pathogenic (PM1, PM2, PM3, PP3)
c.7412G>C	p.(Gly2471Ala)	n/a	n/a	likely pathogenic (PM1, PM2, PM3, PP3)

c.7437-2del	p.?	n/a	n/a	pathogenic (PVS1, PM2, PM3, PP3)

n/a: not listed in the database. homo: homozygous. Het: heterozygous. MAF: minor allele frequency. -: not evaluated. wt: wildtype, mut: mutation. Scores are in brackets.

gnomAD: The Genome Aggregation Database version v2.1.1 (http://gnomad.broadinstitute.org/). The data set provided on this website spans 125,748 exome sequences and 15,708 whole-genome sequences from unrelated individuals sequenced as part of various disease-specific and population genetic studies.
ClinVar: Version december 2023 (https://www.ncbi.nlm.nih.gov/clinvar/).
ACMG: American College of Medical Genetics and Genomics, Richards et al. (2015).
